# Utility of BIS for sedation management during monitored anaesthesia care

**DOI:** 10.4103/0019-5049.65350

**Published:** 2010

**Authors:** Satyen Parida

**Affiliations:** Department of Anaesthesiology and Critical Care, JIPMER, Pondicherry, India

Sir,

I read with interest the article titled “Comparison of Midazolam and Propofol for BIS-Guided Sedation during Regional Anaesthesia” by Khurana *et al*. published in December 2009 issue of IJA.[1] I congratulate the authors on their well-conceived study. However, the comparability of bispectral index (BIS) values between the two stated anaesthetic agents deserves some comment.

Several reports have suggested that BIS measurements may not be independent of the drug used. There is evidence that BIS is influenced by the choice of anaesthetic agent. In a study conducted by Ibrahim *et al*.[2] wherein the BIS values during sedation with sevoflurane, midazolam and propofol were compared, BIS of sevoflurane and propofol patients frequently decreased to less than 60, whereas the BIS of midazolam patients was never less than 65 despite several patients being unresponsive. In addition, mean BIS scores of patients sedated with sevoflurane and ‘responding to name when called repeatedly’ (responders) were significantly different from the BIS scores of midazolam and propofol responders, and BIS scores of propofol nonresponders were significantly different from those of sevoflurane and midazolam nonresponders. They also demonstrated that BIS was a slightly more accurate predictor of depth of sedation with propofol than sevoflurane or midazolam. Mi *et al*.[3] found BIS values at unresponsiveness to voice command were 66 with propofol alone compared with 75 when propofol was administered with fentanyl. Kearse *et al*.[4] found a BIS_50_ (BIS at which 50% of patients were unresponsive) of 65 for propofol alone but 76 for propofol with nitrous oxide. Iselin-Chaves *et al*.[5] reported a BIS_50_ of 64 for propofol alone and 72 for propofol with alfentanil.

As per the study protocol, the initial BIS targeted in both the midazolam and propofol groups was 75, with infusions manipulated intra-operatively to maintain BIS values in the 65–85 range. The incidence of awareness has been stated to be 20% in the midazolam group and 16.7% in the propofol group.

However, in view of the above evidence, identical BIS values may not necessarily indicate identical levels of sedation for both midazolam and propofol as understood in clinical parlance. The authors’ concern that assessment of depth of sedation by clinical means may itself alter such a depth is no doubt valid. Despite such concerns, however, it would have been interesting to note the levels of sedation at identical BIS levels for both groups and infer there from. Furthermore, the methods by which awareness during sedation, as defined by recall of intraoperative events, in the study under discussion were assessed, probably required greater elaboration. In this regard, specific memory tests for immediate and delayed recall as utilized by Ibrahim *et al*.[2] might have helped pick up differences in awareness between the two groups much better.
